# Selective extracellular vesicle exclusion of miR-142-3p by oral cancer cells promotes both internal and extracellular malignant phenotypes

**DOI:** 10.18632/oncotarget.14862

**Published:** 2017-01-27

**Authors:** Christopher T.D. Dickman, James Lawson, James Jabalee, Sara A. MacLellan, Nancy E. LePard, Kevin L. Bennewith, Cathie Garnis

**Affiliations:** ^1^ Department of Integrative Oncology, British Columbia Cancer Research Centre, Vancouver, BC, Canada; ^2^ Department of Pathology and Laboratory Medicine, University of British Columbia, Vancouver, BC, Canada; ^3^ Division of Otolaryngology, Department of Surgery, University of British Columbia, Vancouver, BC, Canada

**Keywords:** MiRNA, exosome, TGFBR1, oral squamous cell carcinoma, oral dysplasia

## Abstract

Packaging of small molecular factors, including miRNAs, into small extracellular vesicles (SEVs) may contribute to malignant phenotypes and facilitate communication between cancer cells and tumor stroma. The process by which some miRNAs are enclosed in SEVs is selective rather than indiscriminate, with selection in part governed by specific miRNA sequences. Herein, we describe the selective packaging and removal via SEVs of four miRNAs (miR-142-3p, miR-150-5p, miR-451a, and miR-223-3p) in a panel of oral dysplasia and oral squamous cell carcinoma cell lines. Inhibition of exosome export protein Rab27A increased intracellular concentration of these miRNA candidates and prevented their exclusion via SEVs. Increased intracellular miR-142-3p specifically was found to target TGFBR1, causing a decrease in TGFBR1 expression in donor cells and a reduction of malignant features such as growth and colony formation. Conversely, increased excretion of miR-142-3p via donor cell SEVs and uptake by recipient endothelial cells was found to reduce TGFBR1 activity and cause tumor-promoting changes in these cells *in vitro* and *in vivo*.

## INTRODUCTION

Extracellular vesicles (EVs) are a heterogeneous group of small membrane bound vesicles that include exosomes, microvesicles, apoptotic blebs and large oncosomes [1–4]. Those vesicles which are smaller than 150 nm regardless of origin are referred to as small EVs (SEVs) [4]. SEVs are known to contain various cargo including proteins, mRNA, and miRNAs [5]. MiRNAs are short, ~22 bp non-coding RNAs that can inhibit the translation of targeted mRNAs. A single miRNA can alter the protein expression of several genes and has been linked to numerous disease processes, including cancer [6–8]. SEVs are increasingly being evaluated as biomarkers for delineating clinical disease states [9–11], as therapeutic targets [12], and as drug delivery vehicles [13]. Further, tumor-derived SEVs have also been reported to act on distant lymph nodes prior to metastasis, creating favorable conditions for angiogenesis and extra-cellular matrix changes that can prime a pre-metastatic niche that promotes subsequent metastatic tumor growth [14, 15].

Packaging of miRNAs into SEVs has been reported by many as selective rather than indiscriminate [16–18]; the mechanisms governing this process are not well understood, though they may involve protein binding to distinct sequence motifs [19–22]. Small RNAs can be enriched in SEVs, with some miRNAs exhibiting much higher enrichment in SEVs as compared to the cells that produced the SEVs (which may not contain detectable intracellular levels of a given SEV enriched miRNA) [16–18]. MiRNA content in SEVs also varies based on cell type and cell state, with miRNA content in SEVs isolated from normal cells in particular differing from miRNA content in dysplastic and cancer cells [9, 23, 24].

The selective packaging of specific miRNAs into SEVs in tumors implies a biological role and can be driven by selection for conditions that promote or inhibit malignancy. Significantly, some SEV-packaged miRNAs have exhibited oncogenic activity, while others demonstrate tumor suppressive functions; for example, in head and neck cancer secreted mir-24-3p, miR-891a, miR-106a-5p, miR-2a-5p, and miR-1908 can decrease T-cell response in the tumor stroma by targeting the Mark1 signaling pathway [25] while secreted miR-21-5p can promote metastasis [26]. SEV secreted miRNAs: miR-150-5p, miR-214-3p, miR-92a-3p and miR-210-3p have been shown to have pro-angiogenic roles [27–30]. Alternatively healthy cells may attempt to attenuate a tumor as demonstrated by immune cells such as macrophages which, can secrete growth inhibitory miR-142-3p and miR-223-3p which target hepatocellular cells [31].

While studies have typically focused on the role of SEV-released miRNAs in cell-cell communication, miRNAs may also be selectively packaged for SEV-mediated release as a means of eliminating a tumor suppressive factor from the cancer cell as has been reported with the miRNAs let-7 in gastric cancer and miR-23b-3p in bladder cancer [32, 33]. It is also possible that a given miRNA might have a dual role in facilitating malignant processes both locally by eliminating tumor suppressive miRNAs and via cell-cell communication as these miRNAs promote angiogenesis when expressed in endothelial cells [34, 35].

This paper reports the miRNAs which, are selectively secreted from oral squamous cell carcinoma cells and oral dysplasia cells. Follow up with one of these miRNAs: miR-142-3p found that secretion of this miRNA from oral cancer cells promotes growth of the cancer cell by eliminating the miRNAs tumor suppressive effect. Released miR-142-3p also affects the tumor microenvironment by promoting angiogenesis which in tumor xenografts leads to decreased hypoxia. Both of these actions are mediated by the protein TGFBR1 a direct target of miR-142-3p.

## RESULTS

### Identification of miRNAs under selection

**Figure 1:**
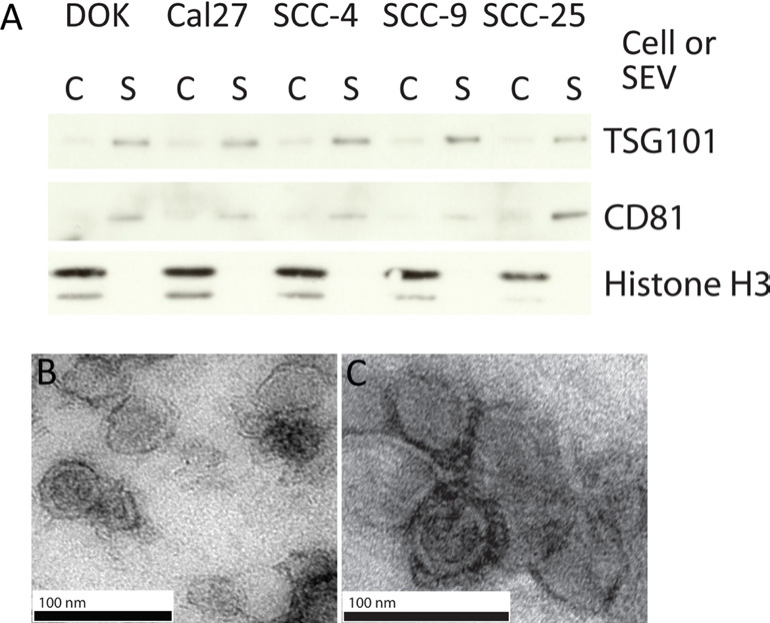
The isolation of SEVs (**A**) Western blot on 10 μg of protein isolated from SEVs for exosome enriched markers TSG101 and CD81; and negative control Histone H3. (C refers to the cellular fraction and S to SEV). (B, C) Uranyl acetate negative stained TEM images, of EVs isolated from SCC-4 (**B**) and Cal27 cell lines (**C**).

**Figure 2:**
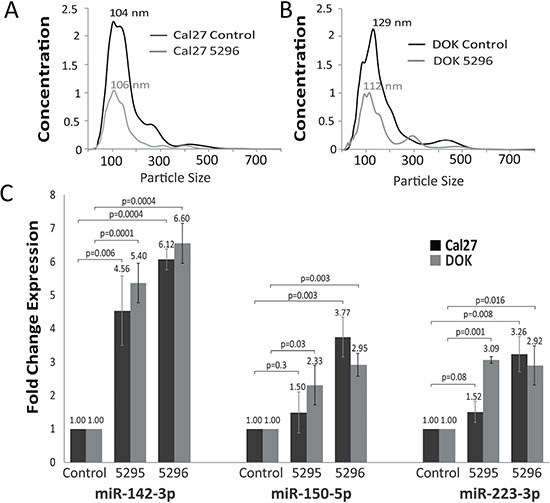
Result of exosome release inhibition (**A**, **B**) NanoSight determined size distribution of particles obtained during SEV precipitation in Cal27 (A) and DOK (B) cells with either control shRNA vector or Rab27A KD 5296 vector. Concentration represents 10^9^ particles per ml. (**C**) Cellular fraction fold change RT-PCR expression values of miR-142-3p, 150-5p and 223-3p in Cal27 and DOK cells after Rab27A knock down compared to control cell lines (*n* = 3) error bars represent standard deviation and *P* values were determined by Student's *t*-test on delta delta CT levels.

To identify miRNAs that are selectively released and/or retained via SEVs in oral cancer and dysplasia cells, SEVs were collected from Cal27, SCC-4, SCC-9, and SCC-25 cancer cell lines and the oral dysplasia cell line DOK by ultra-centrifugation [36]. Western blots for known exosomal proteins CD81 and TSG101, as well as Histone H3 (which is not expected to be present in SEVs) were performed on all SEV extracts and compared to protein levels in donor cells (Figure 1A) [37]. TSG101 and CD81 were increased in the SEV fraction of all samples relative to donor cells, while H3 was strongly expressed in donor cell fractions and only detected at negligible levels in SEV fractions. This suggests that the isolated SEVs were enriched for exosomes with the possible inclusion of other similarly sized vesicles and particles. SEVs were further characterized using transmission electron microscopy (TEM) on isolates from SCC-4 and Cal27 cells (Figure 1B and 1C), and NanoSight on isolates from DOK, Cal27, SCC-4, SCC-9 and SCC-25 cells (Figure 2A and 2B, black lines, and Supplementary Figure 1), with both tests showing a largely homogenous population of vesicles of approximately 100 nm in diameter.

There is substantial discussion on the correct nomenclature of SEVs and what is frequently referred to as ‘exosomes’ in experiments on their functional role, i.e. small EVs derived from ultracentrifugation, may not meet more strict definitions of exosome, in other words endosomally derived small vesicles which may share common protein markers with other vesicles [4, 38]. For the purpose of this paper we will refer to ultracentrifuge derived vesicles as SEVs with the understanding that this is a mixed population of ~100 nm particles, that is enriched for category III EVs including exosomes as defined by Kowal *et al*. [4].

RNA was isolated from all cell lines, as well as matched SEV samples and analyzed using qRT-PCR (Supplementary Table 1). Donor cells exhibited expression of 27–34% of the 742 miRNAs examined, while SEVs expressed 27–39% of these 742 miRNAs. One hundred thirty-one miRNAs were detected in both the donor cell and SEV RNA populations from all five cell lines. Using a 4-fold expression difference threshold to determine enrichment in either donor cells or SEVs, each cell line had an average of ten miRNAs enriched in donor cells and 17 miRNAs enriched in SEVs. Top candidates were selected based on the frequency with which four-fold expression differences were noted. The miRNAs enriched in all SEV samples were: miR-142-3p, miR-150-5p, miR-451a, and miR-223-3p. In addition, miR-126-3p was enriched in 4/5 lines, while miR-126-5p, miR-144-3p, and miR-605-5p were enriched in 3/5 lines. Candidate miRNAs enriched in SEVs were not detectable in the cellular fraction in most instances. The only miRNA candidate enriched in all donor cells was miR-502-5p, which was not detected in any SEV fraction. MiR-197-3p was enriched in 3/5 donor cell fractions, though still detectable in SEVs. The majority of miRNAs detected were not enriched, and instead had similar levels in both the SEV and donor cell fractions (Supplementary Figure 2, Supplementary Table 2).

### Inhibition of SEVs excretion increases cellular miRNA concentration

To determine if miRNA candidates were extracted from SEVs and not co-precipitating factors, we blocked exosome release and assayed for miRNA expression within cells. Multiple known pathways mediate exosome formation and release. One method involves transformation of membrane sphingomyelin to ceramide, creating a vesicle inside a multivesicular body. This pathway is catalyzed by SMPD3 [39]. Downstream, Rab27A mediates transport of multivesicular bodies to plasma membranes and vesicle docking that drives extracellular release of their exosome content [40]. SMPD3 was detectable in only one of the oral dysplasia/cancer cell lines by qRT-PCR (Supplementary Figure 3A), a finding that is consistent with our knowledge of SMPD3 expression in oral tumors [41]. As Rab27A was highly expressed (Supplementary Figure 3A), we silenced it using two different lentiviral shRNAs. In Cal27 cells, knockdown efficiency for shRNA 5295 was 89% and for shRNA 5296 was 96%. In DOK cells, knockdown efficiency for shRNA 5295 was 94% and for shRNA 5296 was 95% (Supplementary Figure 3B). Knockdown of Rab27A led to a decrease in SEV secretion per NanoSight analysis, which also determined that vesicles from Rab27A knockdown and control cell lines were similar in size (Figure 2A and 2B). Silencing Rab27A resulted in an increase in the intracellular content of all candidate miRNAs except for miR-451a, which was not detectable in the cells. Depending on the cell line and shRNA used, miR-150-5p and miR-223-3p exhibited variable increases in expression (Figure 2C). MiR-142-3p was consistently increased > 4-fold intra-cellularly in each cell line following treatment with both Rab27A shRNAs (Figure 2C). SEVs from the Cal27 Rab27A knockdown lines were extracted and profiled for miR-142-3p expression. Knockdown of Rab27A decreased the amount of miR-142-3p excreted from cells by 50.8% and 50.3% with shRNA vectors 5296 (*p* = 0.03) and 5295 (*p* = 0.01)(Supplementary Figure 3C). With the exception of miR-451a these results suggest an association of the candidate miRNAs with exosomes. MiR-451a, may be associated with Rab27A independent exosomes or other vesicular or non-vesicular factors as suggested by others [42, 43].

### TGFBR1 is a target of miR-142-3p

A literature search for potential targets of miR-142-3p using PubMed and GeneRIF [44] revealed TGFBR1 as the only candidate that showed interaction with miR-142-3p in epithelial cancers [45] and has also been implicated in oral cancer progression [45, 46]. These findings are consistent with previous gene expression data showing a decrease in TGFBR1 expression in oral cancer cell lines compared to normal primary lines [47–49]. Additionally it is well established that the 3′UTR of TGFBR1 is capable of binding miR-142 3p [45, 50]

**Figure 3:**
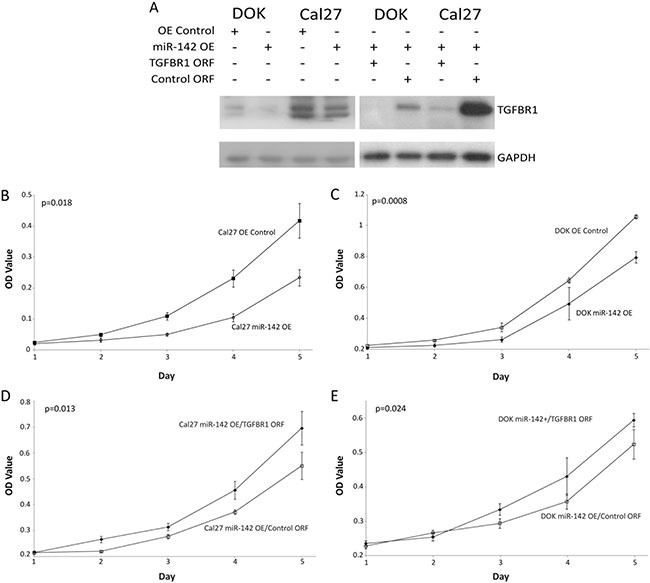
Effects of miR-142-3p over-expression (**A**) Western blot for TGFBR1 levels in DOK and Cal27 with miR-142 OE or OE Control vectors, Percent change values were calculated in ImageJ with levels normalized to GAPDH, and show a decrease in TGFBR1 expression of 70.1% in DOK and 40.0% in Cal27. Additionally Cal27 and DOK miR-142 OE cells were infected with TGFBR1 and control ORF rescue vectors and shown at a lower exposure time. The growth of (B, D) Cal27 and C,E: DOK by MTT proliferation assay, (**B** and **C**) demonstrating the effect of miR-142-3p over-expression and (**D**, **E**) demonstrating phenotypic rescue by the addition of TGFBR1 ORF vector. *P* values were determined by Student's t-test on the final day, error bars represent standard deviation.

To determine if miR-142-3p targets TGFBR1 in OSCC, we stably over-expressed miR-142-3p in Cal27 and DOK cells (creating miR-142 OE lines). To confirm that increased miR-142-3p was excreted via SEVs, SEVs from Cal27 miR-142 OE and Cal27 OE Control cells were collected and qRT-PCR was performed on RNA collected from each cell type. This analysis demonstrated that miR-142-3p was increased 8.71 fold in SEVs collected from miR-142 OE cells as compared with OE Control cells (Supplementary Figure 3D). A western blot for TGFBR1 expression in these cells confirmed a decrease in TGFBR1 expression (Figure 3A). Analysis of western blot results showed that miR-142-3p over-expression was associated with a decrease in TGFBR1 expression by 70.1% in DOK cells and 40.0% in Cal27 cells. This also led to a decrease in the phosphorylation of downstream genes SMAD2 and SMAD3 (Supplementary Figure 3E). Western blots on Cal27 Rab27A KD 5295 and DOK Rab27A KD 5295 showed no effect (not shown) on TGFBR1 expression. Rab27A plays a role in trafficking exosomes to the plasma membrane, this may suggest that miR-142-3p is sequestered within the cell, in exosomes that aren't released.

### MiR-142 decreases the growth rate of oral cell lines

Cal27 and DOK miR-142 OE and OE Control cell lines were tested for the effect of increased miR-142-3p on cellular proliferation using an MTT assay (Figure 3B and 3C). MiR-142-3p had a significant inhibitory effect on the growth of DOK and Cal27, a finding that is consistent with the known role of TFGBR1 [51]. This effect was abrogated by the co-infection of Cal27 and DOK miR-142 OE lines with TGFBR1 ORF clones lacking the 3′UTR binding site of miR-142-3p (Figure 3D and 3E). To analyze the effect of miR-142-3p increase on anchorage independent a colony formation assay was performed on Cal27 cell lines with either the miR-142+ or OE Control vectors (Supplementary Figure 3F). (DOK cells were excluded from this assay, as dysplastic cells do not form colonies.) From three replicates, Cal27 OE Control cells grew 2.8 fold more colonies on average compared to Cal27 miR-142+ (*p* = 0.002). No differences in colony size were noted. Taken together, these data suggest that over-expression of miR-142-3p in oral cancer and dysplasia cells is associated with reduced carcinogenicity *in vitro* at least partially due to by decreasing TGFBR1 expression.

### MiR-142-3p induces angiogenesis *in vitro*

**Figure 4:**
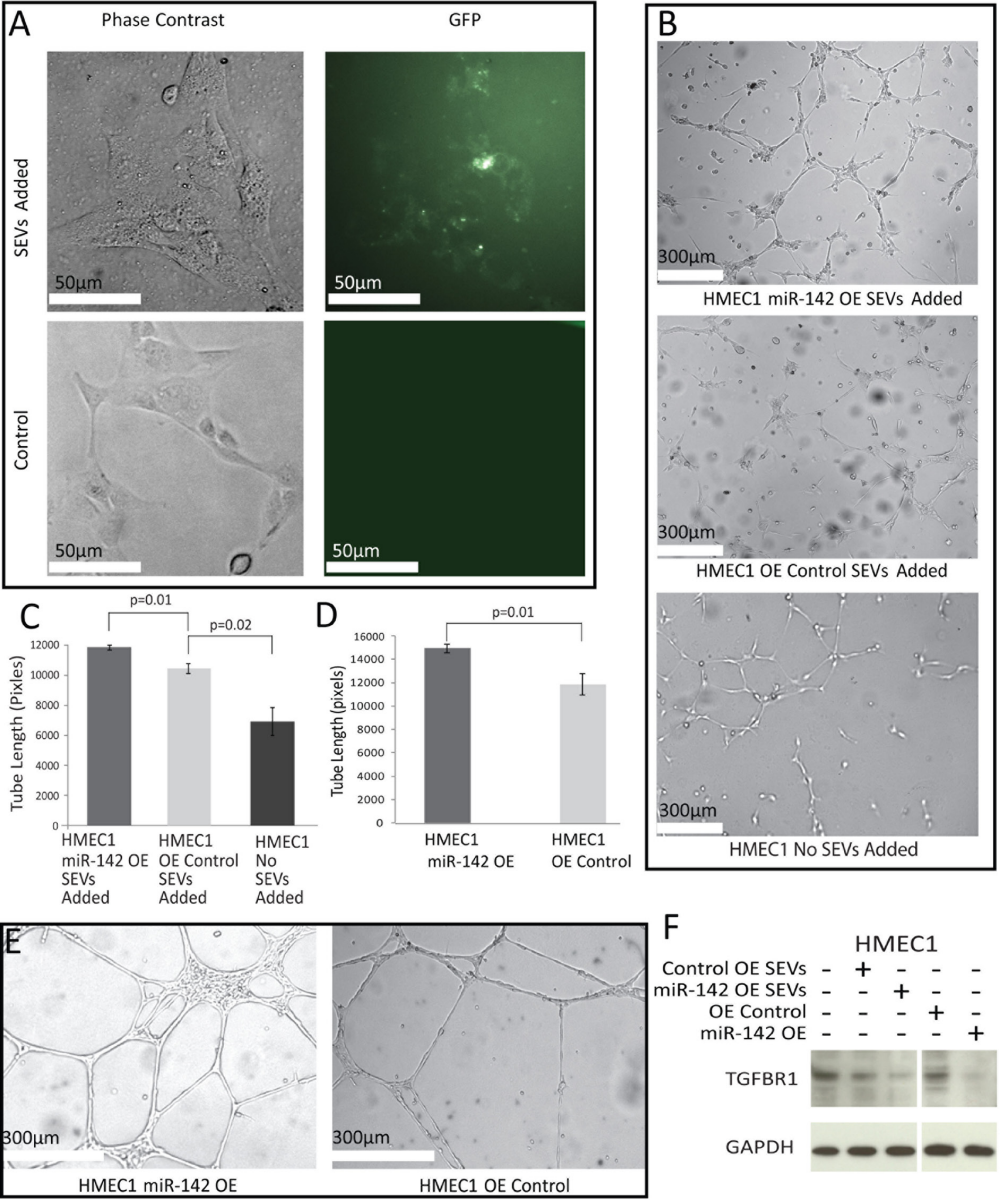
Effects of SEV transfer (**A**) White light and fluorescent images of HMEC1 cells with and without the addition of CD63-GFP stained SEVs under phase contrast. All tube formation assay images were taken 16 hours after seeding. (**B**) Tube formation assay on HMEC1 cells with the addition of SEVs from Cal27 OE Control and Cal27 142 OE. (**C**, **D**) Average length of formed tubes in SEV addition HMEC1 cells (C) or over-expression lines (D) in three fields of view across three wells; error bars represent standard deviation and *p* values were determined by Student's *t*-test. (**E**) Tube formation assay in HMEC1 cells over-expressing miR-142-3p or a control vector. (**F**) Western blot for TGFBR1 and GAPDH comparing the levels in HMEC1 cells after the addition of SEVs extracted from Cal27 OE Control or Cal27 miR-142 OE cell lines and HMEC1 cells expressing miR-142 OE or OE Control vectors. Percent change values were calculated in ImageJ with levels normalized to GAPDH, there was a decrease of 36% and 79% respectively in the levels of TGFBR1 between cells given OE Control SEVs and cells given miR-142 OE SEVs when compared to cells given no SEVs. There was a decrease of 78% TGFBR1 levels between HMEC1 OE Control cells and HMEC1 miR-142 OE cells.

CD63 is associated with intracellular vesicles and SEVs. Fluorescent SEVs from Cal27 cells expressing GFP-labeled CD63 were added to the media of HMEC1 cells growing in 96-well plates and examined under a fluorescent microscope after 48 hours (Figure 4A). Uptake of fluorescent SEVs was observed in the HMEC1 cells in a peri-nuclear fashion.

When expression of TGFBR1 is lower in endothelial cells it has been shown to reduce apoptosis and increase proliferation [52]. *In vitro* tube formation assays are a common surrogate of angiogenesis [53], however it is best to keep in mind the complexity of angiogenic processes, which also involve migration and proliferation [54]. To determine if the SEVs and, more specifically, miR-142-3p within SEVs had an impact on angiogenesis, we added SEVs from Cal27 142 OE and Cal27 OE Control to the media of a tube formation assay performed on HMEC1 cells (Figure 4B). All treatment groups were capable of forming tubes, with miR-142 OE SEVs stimulating development of tubes approximately 1.14 times longer than cells that received SEVs from control Cal27 cells. HMEC1 cells with OE Control SEVs added, developed tubes approximately 1.54 times longer than those observed in cells that were not exposed to SEVs (Figure 4C). The difference in tube formation between HMEC1 cells with Control SEVs and no SEVs is likely due in part by the endogenous amount of miR-142-3p still present in the control SEVs as well as the presence of other factors known to effect angiogenesis such as miR-150-5p [27]. To confirm that miR-142-3p was responsible for at least a proportion of the increase in angiogenesis, the experiment was repeated on HMEC1 cells expressing the miR-142 OE or OE Control vectors with no SEVs added (Figure 4D and 4E). A similar trend was observed with miR-142-3p over-expression causing a significant 1.2-fold increase in tube length relative to HMEC1 cells receiving the OE Control vector. Taken together, these data suggest that miR-142-3p is one factor present in SEVs capable of inducing angiogenesis. The same target of miR-142-3p identified in oral cancer cells, TGFBR1, showed a decrease with the addition of miR-142-3p to endothelial cells both via over expression and the addition of SEVs (Figure 4F).

**Figure 5:**
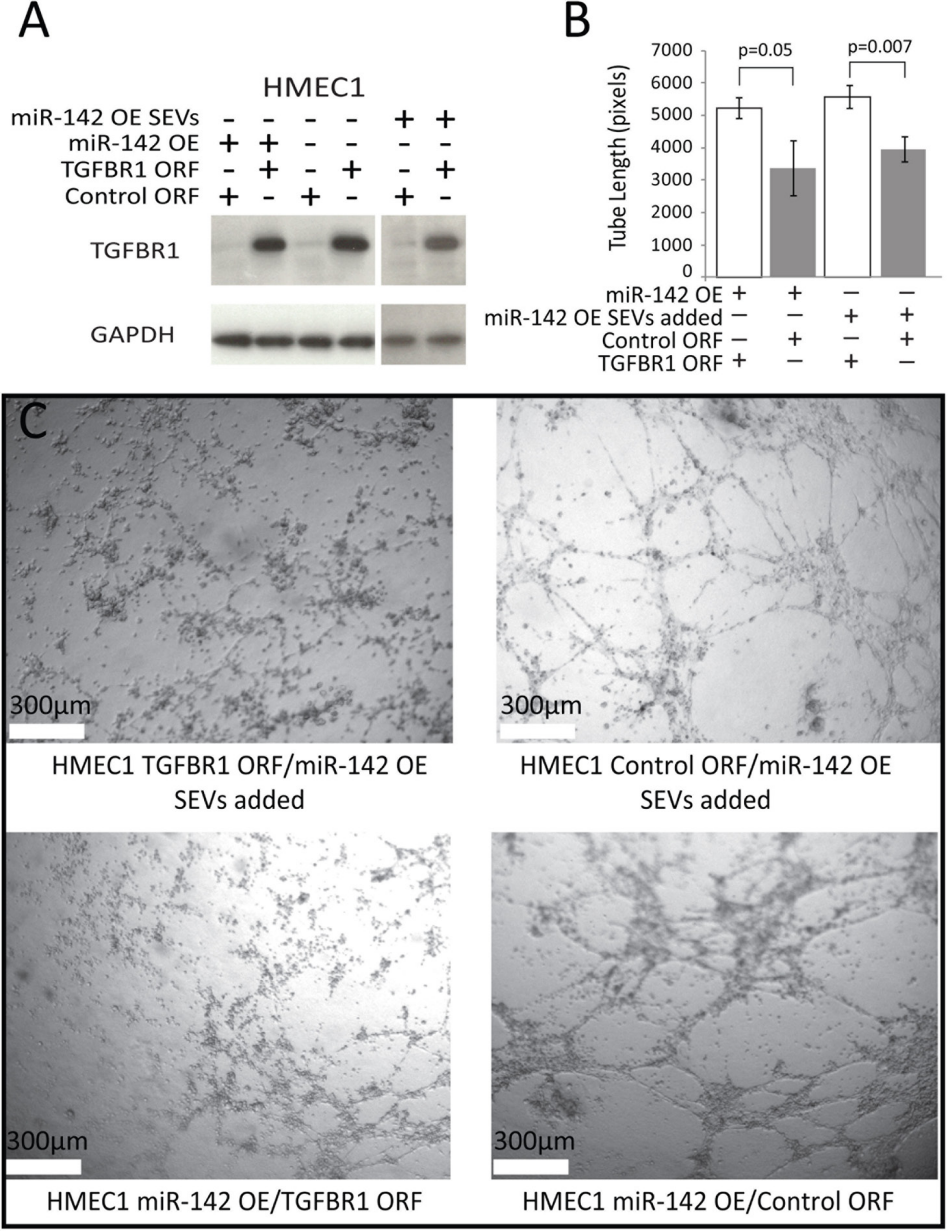
Tube formation phenotypic rescue (**A**) Western blot for TGFBR1, compared to the loading control GAPDH, for HMEC1 cells expressing TGFBR1 ORF or control ORF vectors, with the addition of miR-142-3p from direct over-expression or the addition of SEVs from Cal27 miR-142 OE. (**B**) Average length of tubes formed in HMEC1 cells expressing TGFBR1 ORF or Control ORF rescue vectors, with either co-expression of miR-142 OE vector or addition of Cal27 miR-142 OE SEVs. Error bars represent standard deviation and *P* values were determined by Student's *t*-test. (**C**) Tube formation micrographs of HMEC1 rescue lines.

In order to confirm that alterations to TGFBR1 expression were not incidental to the findings of alterations in tube formation a phenotypic rescue was performed by the addition of exogenous TGFBR1 with an altered 3’UTR, allowing for high TGFBR1 expression in the presences of miR-142-3p (Figure 5A). TGFBR1 over expression caused a decrease in tube formation of 33% in HMEC1 cells over expressing miR-142-3p, and a decrease of 29% when SEVs from Cal27 miR-142 OE were added Figure 5B and 5C).

### Secreted miR-142-3p is associated with increased vascular density *in vivo*

**Figure 6:**
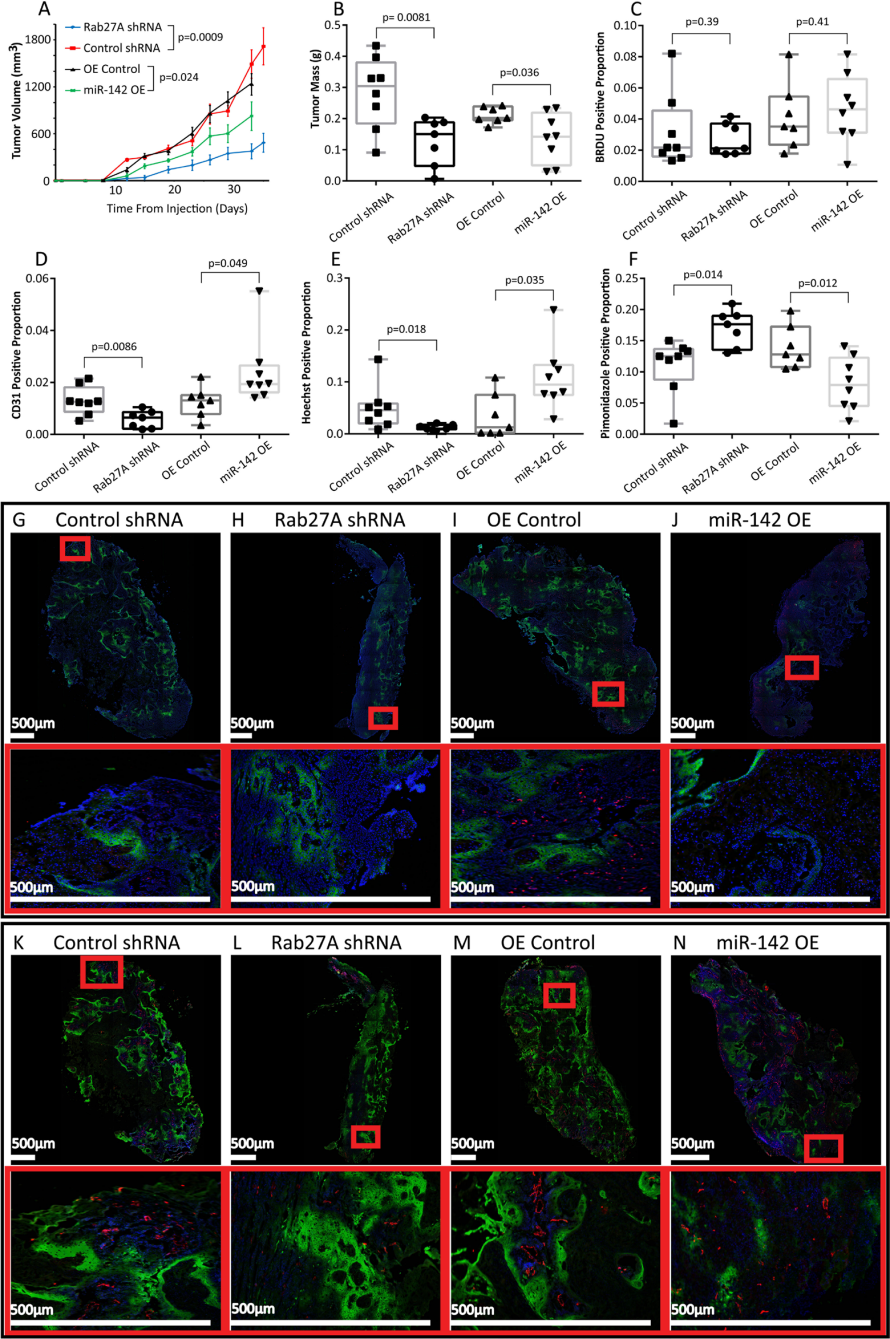
Tumor xenografts (**A**) Average tumor volume over 35 days measured twice weekly in mice injected with 2 million Cal27 cells with Control shRNA (*n* = 8), Rab27A 5296 shRNA (*n* = 7); or the over-expression vectors OE Control (*n* = 7) or miR-142 OE (*n* = 8). Error bars represent standard error. (**B**) Tumor mass taken upon excision on day 35 of Rab27A and Control shRNA tumors and on day 33 of miR-142 OE and OE Control tumors. Box plots showing BRDU positive nuclei relative to all nuclei (**C**); Blood Vessel CD31 staining (**D**) Hoechst perfusion staining (**E**) and Pimonidazole Hypoxia staining (**F**) relative to tumor area. (**A**–**F**) All *p* values were calculated with Student's *t*-test. Tiling images of tumor cross sections, and associated higher magnification images in red boxes. Staining for all nuclei with DAPI (Blue), BRDU (Red), and Pimonidazole (Green) (**G**–**J**) and Injected Hoechst (Blue), CD31 (Red) and Pimonidazole (Green) (**K**–**N**) With Control shRNA (**G**, **K**), Rab27A shRNA (**H**, **L**) OE Control (**I**, **M**) and miR-142 OE (**J**, **N**) tumors.

We were interested in whether SEV secretion and miR-142-3p over-expression affected primary tumor growth and the solid tumor microenvironment. Attempts to inhibit the secretion of only miR-142-3p by MISSION miRNA inhibitors (Sigma-Aldrich) were unsuccessful as the inhibitory effect was not transferred into SEVs and donor cell expression of miR-142-3p was already low due to selective packaging. To determine the effect of reducing SEV production on tumourigenesis, we subcutaneously implanted Rab27A knockdown cells (which reduced secretion of SEVs and as a result, global secreted miRNA content) in SCID mice. Rab27A knockdown tumors showed decreased growth, blood vessel density, and vascular function, and increased tumor hypoxia (Figure 6).

To further elucidate the function of miR-142-3p *in vivo*, we analyzed the impact of miR-142-3p over-expression in mouse xenograft models of oral cancer. Mir-142-3p over-expression significantly decreased the growth rate of implanted tumors (Figure 6A and 6B), consistent with our cell culture findings that indicated that miR-142-3p has an intracellular tumor suppressive effect. We were interested in determining if there was a difference in the proliferative fraction of cells in the primary tumor and therefore measured staining with BrDU, which incorporates into cells in S-Phase. No differences were observed between treatment groups (Figure 6C). Interestingly, miR-142-3p over-expressing tumor xenograft tissues also showed increased vascular density (Figure 6D). The increased number of endothelial cells resulted in an increase in tissue perfusion, suggesting that the blood vessels were functional (Figure 6E). Increased miR-142-3p also led to a decrease in tumor hypoxia (Figure 6F), though hypoxia may be caused by either under-supply or over-consumption of O_2_. This was determined using BrdU a synthetic analog of thymidine which incorporates into DNA that has been replicated in the 90 minutes between injection and euthanasia. No significant differences were observed, which suggests the observed difference in hypoxia was due to blood supply rather than O_2_ consumption, however this assumes the cellular metabolic rate was otherwise similar. These data have shown an increase in functional blood vessels in tumors with over-expression of miR-142-3p but also a decrease in tumor growth.

## DISCUSSION

We sought to determine which miRNAs are selectively secreted from oral cancer cell lines and to determine what function these miRNAs have in promoting carcinogenesis. MiR-142-3p, miR-451a, miR-150-5p, and miR-223-3p were found to be consistently enriched in the SEVs of oral cancer and dysplasia cell lines relative to their expression within the donor cells, complementing previous work showing that miRNAs may be selectively packaged into SEVs in a cell state / cell type-specific manner [9, 23, 24, 27]. These miRNAs have predominantly been shown to be tumor suppressive in cancer cells [45, 55–57], though this does not preclude an oncogenic role in other contexts. For example, SEV-mediated introduction of immune cell inhibiting miR-223-3p and miR-150-5p to tumor stroma may facilitate evasion of immune responses [58, 59].

Secretion of the above miRNAs has been associated with different malignancies: miR-451a and miR-223-3p have been reported as up-regulated in serum samples from esophageal cancer patients [60], and SEVs from monocytes containing miR-150-5p has been reported to promote angiogenesis [27]. These earlier findings, combined with the knowledge that cancer cell lines secrete larger amounts of SEVs than non-cancerous lines [9, 23], suggest that the SEV-mediated miRNA excretion plays a role in promoting the growth of the tumor cell itself, as well as the tumor stroma. These findings align with clinical data showing that high Rab27A expression, and the presumed increase in SEV release that follows, is a poor prognostic indicator in many cancer types [61–63]. Selective retention of miR-502-5p by donor cells may be attributable to an as-yet-undetermined oncogenic function.

MiR-142-3p was selected for follow-up analysis in part because we observed the most consistent increase in its concentration due to shRNA-mediated SEV inhibition (Figure 2C). Most other miRNA candidates exhibited varying increases in concentration due to Rab27A inhibition (Figure 2C), with miR-451a appearing unaffected (a finding that suggests that this miRNA is excreted from the cell in a Rab27A-independent manner). ShRNA-mediated inhibition of Rab27A caused a 12-fold increase in the concentration of miR-142-3p in donor Cal27 cells, while also reducing the amount of SEV-excreted miR-142-3p by approximately half. Since increased miR-142-3p expression in donor cells caused decreased growth (Figure 3A and 3B), this suggests that increased SEV-mediated miR-142-3p secretion may be a means of removing this tumor suppressive effect, a finding that is similar to one reported for miR-23b-3p in bladder cancer cells [33].

Earlier reports have described SEV-secreted miR-150-5p and others as pro-angiogenic factors that mediate cell-cell communication in cell models [27–30]. We have reported SEV-secreted miR-142-3p as an additional pro-angiogenic factor based on our findings that show increase tube formation in endothelial cells (Figure 4). Mouse model experiments showed that miR-142-3p over-expression led to increased functional blood vessel density and associated decreases in hypoxia. This phenotype could have been caused not only by angiogenesis, as suggested by *in vitro* analysis but also vasculogenesis and reduced loss of existing blood vessels. Future research on miR-142-3p's effect on these pathways would be illuminating. It seems as if the blood vessel density promoting effect of miR-142-3p was insufficient to rescue the proliferation decreasing effects of the miRNA, and raises the question of what would happen if only secreted miR-142-3p was increased with no effect on intercellular levels. It is interesting to examine the treatment implications of our results as it is generally expected that tumors with lower levels of hypoxia, are more sensitive to chemotherapy. In OSCC hypoxic tumors are resistant to frontline treatments with 5-fluorouracil and cisplatin. This is mediated by hypoxia induced cell cycle arrest as well as the activation of drug efflux pathways via HIF1a [64, 65]. Vascular density could also increase the availability of reactive oxygen species thereby increasing radiation sensitivity [65].

Significantly, we report that SEV-mediated release of miR-142-3p appears to facilitate multiple malignant processes at the same time; selective exclusion of miR-142-3p mitigates its tumor suppressive growth inhibitory effect in donor oral cancer cells, while also inducing pro-angiogenic activity in recipient cells in associated stroma. Both of these processes involve altered activation of TGFBR1, which mediates TGFB pathway signaling and has been reported to possess both oncogenic and tumor suppressive activity [52, 66, 67]. TGFBR1 and the TGFB pathway in general are known to play a complex role in vascular homeostasis, with both pro- and anti-angiogenic effects. Knockout of TGFBR1 can lead to fragility and malformations in blood vessels while enzymatic inhibition can lead to angiogenesis [68–70]. The angiogenic effects of TGFBR1 appear to be context specific which other researchers have noted increases the difficulty to understanding the pathways molecular action [54, 69]. While this study examined tube formation *in vitro*, and changes in vascular density *in vivo* leading to decreased hypoxia, further work is needed to assess the complete role of TGFBR1 inhibition on the course of OSCC.

*In vivo* analysis on Rab27A inhibition (Figure 6) demonstrates effects on the tumor stroma, opposing the effects of miR-142-3p over-expression. There is a decrease in functional vasculature suggesting that Rab27A plays a tumor promoting role. This is consistent with the hypothesis that the wild type level of secreted miR-142-3p causes increases in blood vessel density and this effect is removed upon Rab27A knockdown. Rab27A knock-down does not affect TGFBR1 levels likely due to the miRNAs being sequestered from the cytosol, and therefore the decrease in growth seen in the Rab27A KD mice is due to the effects of decreased nutrient supply from the limited vascular density. While these results are consistent with a miR-142-3p centered affect, follow up analysis could elucidate an additional role of other secreted miRNAs and proteins, as there are many other associated factors transferred to the stroma in a Rab27A dependant manor. Previous researchers have demonstrated a complementary tumor-promoting effect of Rab27A on metastasis [71, 72]. Future studies will demonstrate the mechanism by which Rab27A promotes vascular density, and tumor cell growth. We also predict there is a functional role of oral SEV signaling to endothelial cells in the formation of pre-metastatic niches, which would complement other's work on the topic [73].

In summary, we have found evidence of selective (rather than indiscriminate) SEV packaging of four miRNAs for exclusion from oral cancer and dysplasia cell lines, as well as selective retention of one miRNA by these same cells. Follow up analysis of one miRNA selectively excluded via SEVs, miR-142-3p, showed that its release leads to both increased growth in donor cells and enhanced tumor supporting potential in cells in associated stroma, both via altered expression of TGFBR1. Further studies building on this work are needed to characterize the machinery driving selective SEV packaging of miRNAs, and to identify the selective forces that alter the behavior of this machinery in the context of malignancy.

## MATERIALS AND METHODS

### Cell lines

The oral cancer cell lines Cal27, SCC-4, SCC-9, and SCC-25 were obtained from ATCC and the oral dysplastic line DOK was obtained from the European Collection of Cell Cultures. Cell lines were maintained according to distributor recommendations. 293T and HMEC1 cells were received as a gift from Dr. Aly Karson and were cultured in Dulbecco's Modified Eagle's Medium supplemented with 10% fetal bovine serum (FBS) at 37°C. RNA was extracted from cells and SEVs using the miRCURY RNA Isolation Kit (Exiqon) per manufacturer instructions.

### SEV isolation

Cell lines were seeded into eight, 15 cm plates. Forty-eight hours before reaching 90% confluency, media was replaced with media containing 1% FBS without noticeably affecting growth. The FBS was depleted of SEVs by ultracentrifugation at 110,000 g overnight in order to reduce contamination from bovine SEVs. Cells were allowed to excrete SEVs into media for 48 hours, after which the conditioned media was collected and subjected to multiple rounds of centrifugation as previously described [36]. Dead cells and cellular fragments were removed from media with 4°C centrifugation at 200 g for 10 minutes, 2,000 g for 30 minutes, and 10,000 g for 60 minutes, with the precipitate being discarded at each interval. SEVs were precipitated using an ultracentrifuge at 110,000 g at 4°C for 70 minutes, after which the supernatant was removed and the pellet was rinsed with PBS. SEVs were re-precipitated with an additional 110,000 g spin for 70 min. RNA was extracted from both SEVs and cells from one of the plates excreting SEVs as described above. Previous researcher has suggested this method might lead to contamination from bovine miRNA not removed by EV depletion [74, 75], and therefore the EV isolation procedure was performed on non-conditioned media with 1% depleted FBS. Supplementary Figure 4 demonstrates a decrease in miRNA below the threshold of detection. Transmission electron microscopy (TEM) images were prepared by absorbing a PBS-suspended sample on 200 mesh formvar-coated nickel grids, allowing the sample to dry, and then fixing the sample by floating the grid on a drop of 1% glutaraldehyde for 10 min. Negative staining was performed by floating the grid on a drop of 1% Uranyl acetate for 1 min. Grids were viewed on a Tecnai 12 microscope.

### Western blotting

Protein was collected through lysis with radioimmunoprecipitation assay (RIPA) buffer supplemented with 1:100 protease inhibitor (Life Technologies) and phosphatase inhibitor cocktail I & II (Sigma-Aldrich). Protein was quantified using a Bicinchoninic Acid Protein assay kit (Life Technologies). 10 μg of protein was separated using NuPAGE 4–12% Bis-Tris Gels (Life Technologies) and transferred to polyvinylidene difluoride membranes (Millipore). Membranes were blocked in 5% BSA, 1X TBS, and 0.1% Tween-20 at room temperature for 1 hour. Membranes were then incubated overnight at 4°C with the appropriate primary antibody: 1:1000 diluted anti-CD63 (EXOAB-Cd63A-1, System Biosciences), anti-TSG101 (ab83, Abcam) or anti-phospoSMAD2/3 (8828 Cell Signaling) antibodies or 1:2000 diluted anti-TGFBR1 (AF3025, R&D Systems).

After washing the antibodies were incubated with peroxidase-conjugated 1:2000 Anti-Rabbit (7074, Cell Signaling Technology), Anti-Mouse (NXA931, GE Healthcare), or Anti-Goat (6741, Abcam). For TGFBR1, anti-GAPDH (1:4000) (2118, Cell Signaling Technology) was used as a loading control. Detection was performed using Amersham ECL Western Blotting Detection Kit (GE Healthcare). To quantitatively assess western blot images of TGFBR1 expression, the ‘gel analysis’ function in the ImageJ program (http://imagej.nih.gov) was used and normalized to GAPDH expression.

### Real time PCR

QRT-PCR was used to identify miRNAs differentially expressed in cells as compared with SEVs. Reverse transcription was performed on 40 ng of RNA using an Exiqon Universal cDNA synthesis kit, and qRT-PCR was performed using Exiqon's SYBR based microRNA Ready-to-Use PCR, Human Panel I + II V2.M as previously described [47] to test for the expression of 742 miRNAs. Follow-up analysis on individual miRNAs was performed on 100 ng of RNA using TaqMan miRNA reverse transcription kits and TaqMan Universal Master Mix II and MicroRNA assays for qRT-PCR (Life Technologies). All assays were performed in triplicate per manufacturer protocol. TaqMan High-Capacity cDNA reverse transcription kits, Gene Expression Master Mix, and Gene Expression Assays (Life Technologies) were used to assess impacts of SMPD3 and Rab27A knockdown.

### Statistical analysis of miRNA data

Pre-processing of SYBR miRNA qRT-PCR data was completed as previously described [7]. We performed a paired analysis to identify miRNAs that were differentially expressed between SEVs and their parental cell lines (i.e. the cells that gave rise to them). MiRNAs were considered enriched if 1) a > 4-fold difference in expression was noted between SEV and cellular fractions following normalization to the global mean of miRNA expression [76] or 2) a given miRNA was detected in one fraction (either cell or SEV) at a CT below 33 and not detected (at a CT below the threshold of detection: 35) in the matched fraction for all of the cell lines tested.

For follow-up miRNA analysis, cellular miRNA quantification was normalized to the expression of U6. As there is no known endogenous control for SEV miRNAs, follow-up quantification was performed with expression normalized to the cel-miR-39 spike-in control (Qiagen). Analysis of knockdown efficiency of SMPD3 and Rab27A expression was normalized to GAPDH.

### Over-expression and shRNA vectors

Over-expression FIV lenti-vectors were purchased from GeneCopoeia for miR-142-3p (HmiR02082-MR01) and a scramble sequence control (CmiR0001-MR01) (hereafter miR-142 OE and OE Control). The miR-142 OE vector produces both 3p and 5p strands. Vectors were packaged using 293T cells and a GeneCopoeia Lenti-Pac FIV Expression Packaging Kit (FPK-LvTR) according to the manufacturer's recommendations. For knockdown of Rab27A, shRNA vectors were purchased from Dharmacon. Five vectors were purchased to determine knockdown efficiency (TRCN0000005294, TRCN0000005295, TRCN0000005296, TRCN0000005297, TRCN0000005298) the 2 vectors with the highest efficiency were used for ongoing analysis (TRCN0000005295, TRCN0000005296 hereafter 5295 and 5296 respectively). Empty pLKO.1 was used as a control. SEV staining vector pCT-CD63-GFP was purchased from SystemBio. For rescue experiments a TGFBR1 open reading frame (ORF) clone (TRCN0000488036) within a pLX_TRC317 vector as well as an empty vector control were purchased from Sigma Aldrich (hereafter TGFBR1 ORF and Control ORF). Lentivirus was created using 293T cells with each shRNA, GFP or ORF plasmid, packaging plasmids VSVG and d8.91 using TransIT-LT1 transfection reagent (Mirus). For all vectors, virus-containing conditioned media was collected over 3 days post transfection and filtered using 0.45 μm filters and stored at −80°C. Cells were selected over 10 days using either 400 μg/mL G418 (over-expression lines) or 2 μg/mL puromycin (shRNA, ORF and GFP lines).

### MTT cell proliferation assay

Stably infected miR-142-3p OE cells and their controls were plated in 6 wells of five 96 well plates at a density of 1000 cells for DOK infected with either miR-142 OE, OE control, or combined miR-142 OE/TGFBR1 ORF, or miR-142 OE/Control OFF. 500 Cal27 cells infected with the same vectors were plated with 500 cells per well. Cell viability was measured once a day for 5 days using Colorimetric thiazolyl blue tetrazolium bromide (Sigma-Aldrich) as previously described [77] with the highest and lowest value from each condition being discarded. Statistical significance was determined using Student's *t*-test on day 5 with a cutoff value of *P* < 0.05.

### Soft agar colony formation assay

A 12-well plate was filled with a bottom layer of 0.5% low melting point (LMP) agarose. A layer made with 2000 cells from both Cal27 over-expression Control and Cal27 miR-142+ lines in 0.37% LMP agarose was added on top of the bottom layer. After four weeks the number of colonies per well was counted for both lines. All experiments were performed in triplicate. Colonies which consisted of at least 15 cells were counted.

### Tube formation assay

HMEC1 cells were grown on a 96 well plate and upon reaching 50% confluency SEVs were added to the cell culture media. SEVs were isolated from one, 15mm plate of Cal27 CD63-GFP cells and diluted in 100μl of DMEM media. This was performed in triplicate. HMEC1 cells were grown in SEV containing media for 24 hours after which the media was replaced with un-modified DMEM. White and fluorescent images were taken using phase contrast on an Axiovert S1000 microscope. This was also performed with SEVs isolated from Cal27 142 OE or Cal27 OE Control with RNA being extracted after 24 hours.

Tube formation assays were performed by the addition of 100 μL of growth factor reduced Matrigel (Corning) at 10 mg/mL to each well of a 24 well plate. The day before the assay HMEC1 cells were grown to 80% confluency then serum starved over night. Three wells of 4.56 × 10^3^ HMEC1 cells were plated in 100 μL DMEM supplemented with 1% FBS on matrigel plates. SEVs isolated from one, 15mm plate of Cal27 142 OE or Cal27 OE Control and diluted in 10μl of PBS were added to the cell culture medium. As a control 10μl PBS was also used. This was performed in triplicate for each treatment group. To test the effect of the miRNA in isolation HMEC1 142 OE or HMEC1 OE Control cells were plated using the same conditions without the addition of SEVs. All tube formation experiments were repeated with HMEC1 cells infected with TGFBR1 ORF or Control ORF vectors to determine the contribution of TGFBR1 to the observed phenotype. Images were taken after 16 hours using differential interference contrast. The average tube length among three replicates was calculated using the ImageJ macro ‘Angiogenesis Analyzer’.

### Tumor xenografts

Immunocompromised NOD-SCID mice were bred and housed in the Animal Resource Center at the BC Cancer Research Center under pathogen free conditions. All experiments were performed in accordance with UBC and Canadian Council on Animal Care guidelines. Male mice were injected subcutaneously with 2 million Cal27 cells infected with either Rab27A shRNA (*n* = 7), Control shRNA (*n* = 8), miR-142 OE (*n* = 8) or OE Control (*n* = 7) vectors. After injection, caliper measurements of tumors were taken twice a week. Volume was calculated as a half ellipsoid using the formula length × width × height × (4π/6). Mice were euthanized on post-injection day 33 (Rab27A shRNA and Control shRNA tumors) or day 35 (miR-142 OE or OE Control tumors). Ninety minutes prior to euthanasia, mice were injected with Intraperitoneal pimonidazole (PIMO) (100 mg/kg), which binds to hypoxic cells, and bromodeoxyuridine (BrdU) (90 mg/kg), which incorporates into replicating DNA and 10 minutes prior to euthanasia with intravenous Hoechst 33342 (Hoechst) (25 μg/mouse) to demarcate perfused vasculature. Tumors were resected, weighed, and then bisected along the sagittal plane and frozen in Optimal Cutting Temperature compound.

### Immunofluorescence

10 μm serial sections were taken from the center of each tumor. Sections were stained with endothelial cell marker 1:200 anti-CD31 (553370, BD Pharmingen) for 90 minutes followed by 1:100 Alexa Fluor 594 conjugated secondary antibody (A-11007, ThermoFisher) for 30 min, and then 1:1000 FITC conjugated anti-PIMO (4.3.11.3, Hypoxyprobe Inc) for 90 min. Subsequent sections were incubated in 2M HCl/0.1% Triton X-100 for 10 min to denature DNA and expose BrdU, then triple stained in sequence with 1:100 anti-BrdU (ab6326, Abcam) for 90 min, 1:100 Alexa Fluor 594-conjugated secondary antibody for 30 min, 1:1000 FITC-conjugated anti-PIMO for 90 min, and 0.1 μg/mL DAPI for 5 min. All slides were mounted with Vectashield (H-1000, Vector Laboratories). Acid washing removed Hoechst staining, and removed background green fluorescence, therefore perfusion was quantified from the first set of slides and hypoxic PIMO staining was quantified from the second set of slides. Whole cross-section tiling images were taken under 10X objective magnification with a Zeiss Imager Z1 using a QImaging Retiga 4000R camera with Northern Eclipse software and staining was quantified using ImageJ as previously described [78]. Images were captured in black and white for each emission spectrum and then combined in false color.

## SUPPLEMENTARY MATERIALS FIGURES AND TABLES




