# Supplementary material for: Selective extracellular vesicle exclusion of miR-142-3p by oral cancer cells promotes both internal and extracellular malignant phenotypes
Source: Oncotarget. 2017 Jan 27;8(9):15252–66. doi: 10.18632/oncotarget.14862 (PMC5362484; doi:10.18632/oncotarget.14862)
Supplement: Supplementary file 1 [file oncotarget-08-15252-s001.pdf]

Selective extracellular vesicle exclusion of miR-142-3p by oral cancer cells promotes both internal and extracellular malignant phenotypes

Supplementary Materials

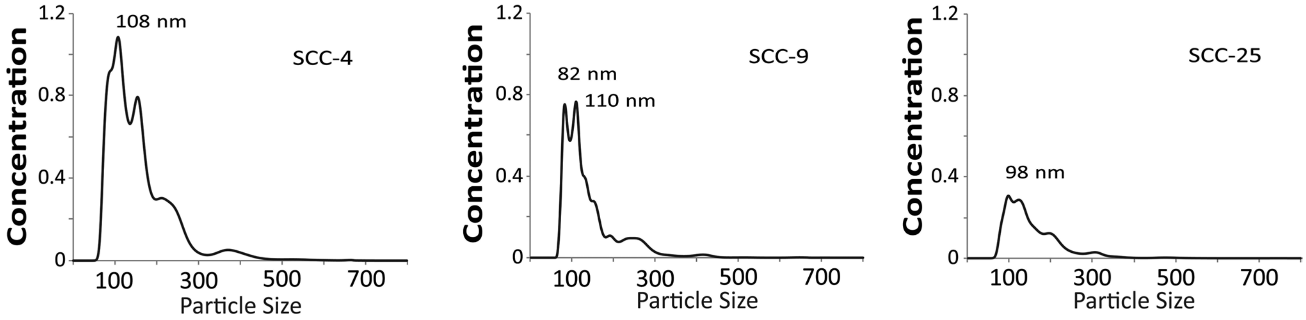

Supplementary Figure 1: NanoSight determined size distribution of particles obtained during SEV precipitation in SCC-4, SCC-9, and SCC-25 cells. Y access denotes particles $\times 10^9$ per ml and was normalized to 100,000 secreting cells.

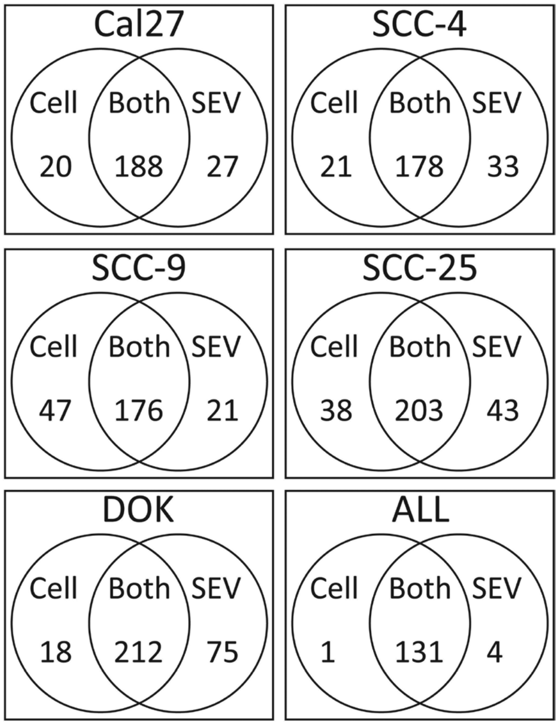

Supplementary Figure 2: Venn diagrams showing the number of miRNAs detected below a CT of 35, for each cell line by qRT-PCR, and what fraction the miRNAs were detected in.

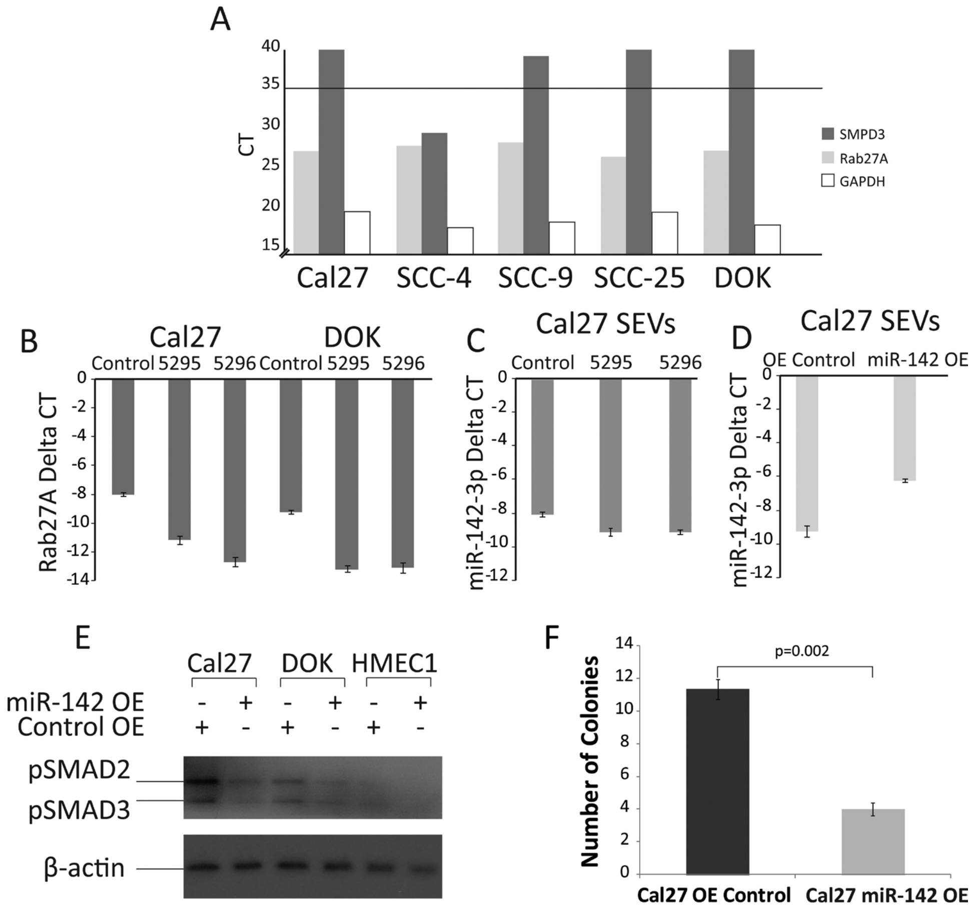

Supplementary Figure 3: Analysis of knockdown and over-expression cells. (**A**) shows qRT-PCR CT values of the endogenous quantities of the SMPD3 and Rab27A, The black line represents our threshold of detection above which there may be amplification in no template controls. (**B**) shows the knockdown efficiency of Rab27A shRNAs in Cal27 and DOK. GAPDH was used to normalize (**C**) The amount of miR-142-3p secreted from the cells after SEV release was inhibited by Rab27A, Equal volumes of RNA were loaded and normalized to cel-miR-39 spike-in. (**D**) shows the amount of miR-142-3p over-expression after lenti-infection in the SEVs collected from Cal27 cells. Error bars represent the standard deviation over three replicates spike-in cel-miR-39 was used to normalize. (**E**) Western blot of pSMAD2/3 on cell lines with miR-142-3p over-expression. (**F**) Colony formation assay, showing average number of colonies across three wells after 5 weeks, p values represent student's *t*-test and error bars represent standard deviation.

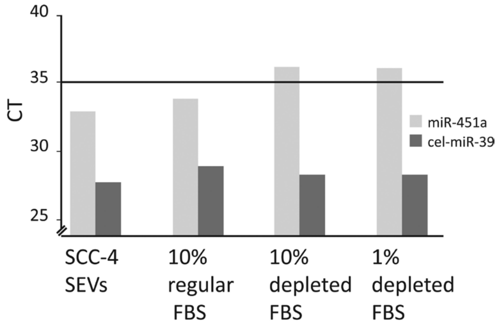

Supplementary Figure 4: Effects of SEV depletion of FBS. qRT-PCR results of RNA isolated from SCC-4 SEVs and blank media supplemented with 10% regular FBS, 10% depleted FBS or 1% depleted FBS. miR-451a was tested along with spike in cel-miR-39. The black bar represents the threshold of detection above which there may be no template control amplification. Equal volumes of miRNA were run using TaqMan qRT-PCR.

Supplementary Table 1: miRNA expression data for cell lines and matched SEVs. See Supplementary_Table_1

Supplementary Table 2: miRNAs and the cellular or extracellular fraction they are detected in. See Supplementary_Table_2
